# Emergence and Polyclonal Dissemination of *bla*_NDM-7_–Carrying InX3 Plasmid in *Enterobacter cloacae* Complex, France, 2021–2023

**DOI:** 10.3201/eid3110.250830

**Published:** 2025-10

**Authors:** Ines Rezzoug, Delphine Girlich, Aurelien Birer, Richard Bonnet, Josephine Poiraud, Pierre Bogaerts, Cécile Emeraud, Laurent Dortet

**Affiliations:** Author affiliations: Université Paris-Saclay, Fontenay-aux-Roses and Le Kremlin-Bicêtre, France (I. Rezzoug, D. Girlich, C. Emeraud, L. Dortet); Assistance Publique-Hôpitaux de Paris, Le Kremlin-Bicêtre (I. Rezzoug, J. Poiraud, L. Dortet); Associated French National Reference Center for Antibiotic Resistance: Carbapenemase-Producing Enterobacteriaceae, Le Kremlin-Bicêtre (I. Rezzoug, C. Emeraud, L. Dortet); Microbes, Intestin, Inflammation et Susceptibilité de l'Hôte, Clermont-Ferrand, France (A. Birer, R. Bonnet); Centre National de Référence de la Résistance aux antibiotiques, service de Bactériologie, CHU Gabriel-Montpied, Clermont-Ferrand (A. Birer, R. Bonnet); National Reference Laboratory for Monitoring of Antimicrobial Resistance in Gram-Negative Bacteria, CHU Dinant-Godinne, UCL Namur, Yvoir, Belgium (P. Bogaerts)

**Keywords:** Enterobacter cloacae, bacteria, antimicrobial resistance, carbapenemases, NDM, New Delhi metallo-β-lactamase, epidemiology, Enterobacterales, β-lactamase, France

## Abstract

Among 3,367 New Delhi metallo-β-lactamase–producing Enterobacterales isolates collected in France during 2021–2023, we found the *bla*_NDM-7_ gene systematically localized on 2 closely related InX3 plasmids known to harbor antimicrobial resistance and virulence factors. Enhanced surveillance to monitor spread of antimicrobial resistance is needed among New Delhi metallo-β-lactamase–producing Enterobacterales.

Carbapenems are among the last-resort antimicrobial agents available to treat infections caused by multidrug-resistant gram-negative bacteria. Extensive use of carbapenems has led to emergence of carbapenem-hydrolyzing enzymes, known as carbapenemases, that hamper last-resort antimicrobial drug therapies (*1*). Carbapenemase-encoding genes are mostly carried on plasmids, which enable rapid dissemination of those genes among gram-negative bacteria.

In France, OXA-48–like enzymes are the most prevalent carbapenemases in Enterobacterales (*2*). However, in recent years, the prevalence of metallo-β-lactamases, particularly New Delhi metallo-β-lactamase (NDM), increased exponentially (*3*), and 86 NDM variants had been identified by April 2025 (http://www.bldb.eu). Diverse plasmid types, such as IncF, IncA/C, IncL/M, IncH, IncN, and IncX3, carry *bla*_NDM_ genes (*3*,*4*). We characterized NDM-producing Enterobacterales circulating in France during 2021–2023 to decipher the prevalence of NDM variants among Enterobacterales species.

## The Study

**Figure 1:**
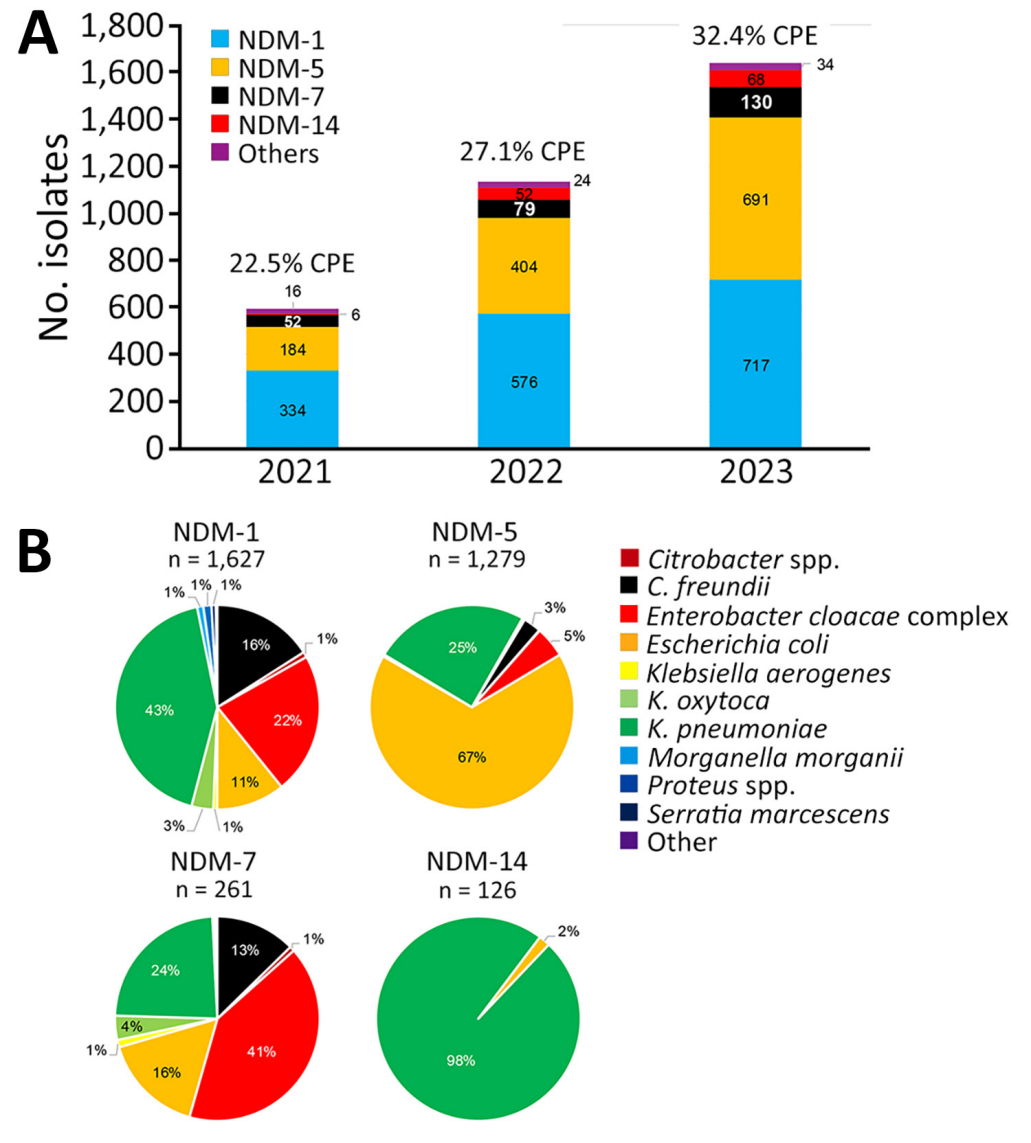
Variants and species involved in emergence and polyclonal dissemination of *bla*_NDM-7_–carrying InX3 plasmid in *Enterobacter cloacae* complex, France, 2021–2023. A) Distribution of NDM variants among CPE. B) Enterobacterales species distribution for the 4 most prevalent NDM variants detected. CPE, carbapenemase-producing Enterobacterales; NDM, New Delhi metallo-β-lactamase.

Among 11,825 carbapenemase-producing Enterobacterales (CPE) isolates received at the French National Reference Center for Antimicrobial Resistance (F-NRC) during January 1, 2021–December 31, 2023, we recovered and genetically characterized 3,367 NDM-producing isolates (Appendix 1). We found that the number of NDM producers increased from 22.5% (592/2,582) of all CPE isolates in 2021 to 27.1% (1,135/4,187) in 2022 and 32.4% (1,640/5,056) in 2023 (Figure 1, panel A; Appendix 2). Among isolates, NDM-1, NDM-5, NDM-7, and NDM-14 represented 98% of all NDM enzymes (Figure 1, panel B). 

We identified NDM-type carbapenemases in 10 different genera (Appendix 1), but the distribution of NDM variants varied drastically within Enterobacterales species (Figure 1, panel B). We identified NDM-1 mainly in *Klebsiella pneumoniae* (43%) and NDM-5 predominantly in *Escherichia coli* (67%), and we found that NDM-14 was nearly exclusively associated with *K. pneumoniae* (98%). Unexpectedly, *Enterobacter cloacae* complex (ECC) accounted for 41% (107/261) of the NDM-7 producers.

**Figure 2:**
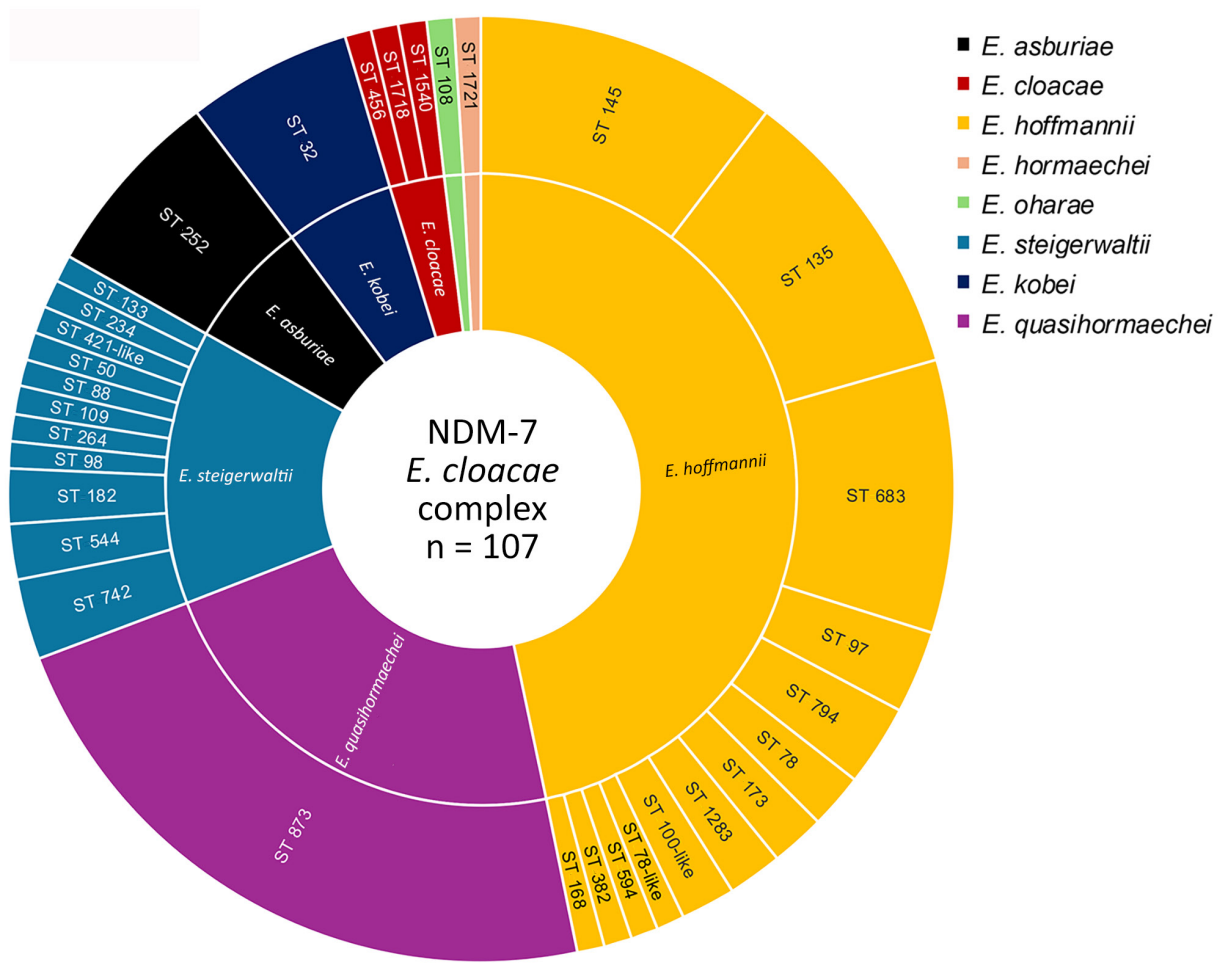
Distribution of STs among 107 *Enterobacter cloacae* complex isolates used in a study of emergence and polyclonal dissemination of *bla*_NDM-7_–carrying InX3 plasmid, France, 2021–2023. ST, sequence type.

Among the 107 NDM-7–producing ECC isolates, we identified 32 different sequence types (STs), the most prevalent of which were ST873 (22.4%), ST135 (10.2%), ST145 (10.2%), ST683 (9.3%), ST252 (6.5%), and ST32 (5.6%) (Figure 2). Those STs corresponded to 8 different *Enterobacter* species, including 46.7% of *E. hormaechei* subspecies *hoffmanii* (mostly ST135, ST145, and ST683), 22.4% *E. quasihormaechei* (all ST873), 7.4% *E. hormaechei* subsp. *steigerwaltii*, 6.5% (7/107) *E. xianfangensis*, 6.5% *E. asburiae*, 5.6% *E. kobei* (all ST32), 2.8% *E. cloacae*, 0.9% *E. hormaechei* subsp. *oharae*, and 0.9% *E. hormaechei* subsp. *hormaechei*. Phylogenetic analysis confirmed the polyclonal dissemination of NDM-7–producing ECC, including in the 4 major STs (ST873, ST145, ST135, and ST683) comprising several isolates (Appendix 1 Figure 1).

Among the 107 NDM-7–producing ECC isolates, short-read sequencing enabled us to identify 12 different plasmid replicases (Appendix 2). Long-read sequencing performed on 30 representative NDM-7–producing ECC showed that *bla*_NDM-7_ was carried on an IncX3-type plasmid in all isolates (Appendix 1). Of note, 93.3% (28/30) of the IncX3 plasmids were 46,161-bp long, and 6.7% (2/30) were 49,830-bp long (Appendix 1 Figure 2). The size difference corresponded to the Tn*5403* transposon. Those 2 plasmids shared 99.9% nucleotide identity with the 46,161-bp *bla*_NDM-5_–carrying plasmid pEC21Z078-46K (GenBank accession no. CP10126), previously described in an *E. coli* isolate from China (*5*).

To compare the *bla*_NDM-7_–carrying plasmids with those harboring *bla*_NDM-5_ in *Enterobacter* spp., we performed long-read sequencing on 19 additional NDM-5–ECC isolates (Appendix 2). We found that 96% of those isolates also harbored an IncX3 plasmid. Among them, 84.2% (16/19) harbored an IncX3 plasmid almost identical to one of the *bla*_NDM-7_–carrying plasmids (except the 2 single-point mutations between *bla*_NDM-5_ and *bl*a_NDM-7_); 12 harbored the 46,161-bp plasmid and 4 harbored the 49,830-bp plasmid. Among the other 3 NDM-5–producing ECC isolates, we localized *bla*_NDM-5_ on different IncFII-type plasmids: a 177,571-bp and a 96,517-bp plasmid similar to pABC143C-NDM (GenBank accession no. KY130431.1), and a 187,303-bp plasmid similar to p60214CZ (GenBank accession no. CP085746.1).

To investigate the origin of the *bla*_NDM-7_ IncX3 plasmid, we further explored 79 full-length sequences of NDM-1–producing ECC for IncX3 plasmids. Among the 11 different replicases identified, IncX3 was in only 14% of NDM-1–producing ECC. Long-read whole-genome sequencing of those 11 IncX3-positive NDM-1–producing ECC identified the *bla*_NDM-1_ gene on IncX3 plasmids of 39,582-bp (n = 7) and 44,682-bp (n = 2) length (Appendix 1 Figure 2). Both of those plasmids were close to *bla*_NDM-5_– and *bla*_NDM-7_–carrying IncX3 plasmids but showed notable differences in the genetic region surrounding the *bla*_NDM_ gene (Appendix 1 Figure 2). Despite detection of an InX3 plasmid, the other 2 strains harbored *bla*_NDM-1_, either directly localized on the chromosome or on a 51,088-bp IncFII plasmid that had no similarity to plasmids available in GenBank.

The close genetic context of *bla*_NDM_ genes localized on IncX3 plasmids showed that both *bla*_NDM-7_ and *bla*_NDM-5_ were flanked upstream by ΔTn*2*–ΔIS*3000*–IS*Aba125*–IS*5* and downstream by genes encoding *ble*_MBL_, PAI, *ccdA*, IS*26*, UmuD protein, IS*Kox3*, a resolvase encoding gene, and Tn*5403* (Appendix 1 Figure 2). Several elements were truncated or absent in the *bla*_NDM-1_ genetic environment compared with *bla*_NDM-5_ and *bla*_NDM-7_, including truncation of IS*Aba125* and deletions of IS*5*, IS*Kox*3, and Tn*5403*.

Finally, to decipher whether the IncX3 plasmids were more prevalent in ECC, we looked for IncX3 replicase in 20,028 multidrug-resistant Enterobacterales genomes in the F-NRC database collected since 2022. Of those, 11.9% (n = 2,393) of isolates carried the IncX3-encoding replicase gene, 27.5% (n = 658) of which were *Klebsiella* spp., 27.3% (n = 653) were *Citrobacter* spp., 26.8% (n = 642) were *E. coli*, and 17.7% (n = 423) were ECC (Appendix 1 Figure 3).

## Conclusions

During 2021–2023, F-NRC received increasing numbers of carbapenem-resistant Enterobacterales isolates: 2,582 in 2021, 4,187 in 2022, and 5,056 in 2023. In addition, the number of NDM-producing isolates more than tripled during that timeframe (Figure 1).

Although >86 NDM variants have been reported globally in the Beta-Lactamase DataBase (http://bldb.eu), 4 main variants are dominant in Europe: NDM-1, NDM-5, NDM-7, and NDM-14. In France, our results demonstrated that NDM-1 remains predominantly associated to *K. pneumoniae*, among which ST147 has been described as highly prevalent (48%) (*6*,*7*). The *bla*_NDM-1_ gene is carried on various plasmid types, including IncX3 (*8*), IncFIB, IncHI1B, and IncL, that harbor both resistance and virulence factors (*9*). In France, NDM-1 producers have been progressively replaced by NDM-5 producers since 2023. NDM-5 and NDM-7 differ from NDM-1 by substitutions responsible for an enhanced carbapenemase activity (*7*). In Europe, dissemination of NDM-5 is mostly caused by *E. coli* (*10*), which represented 70% of NDM-5 producers in our study. As previously described (*11*), our results confirmed that *bla*_NDM-5_ gene dissemination is mainly mediated by IncX3 plasmids. Our results confirmed that NDM-14–producing *K. pneumoniae* ST147 became established after it emerged in France in 2022 (*12*).

Our results highlight the emergence of NDM-7 in ECC, as noted in 41% of the isolates in our study. However, we did not identify any exclusive or particularly dominant clone involved in the dissemination of NDM-7 ECC, except ST873, which was slightly overrepresented. Of note, ECC isolates have been reported to trigger dissemination of Verona-integron–encoded metallo-β-lactamases in France, of which ST893 is the most prevalent ST (*13*). Our results also raise concerns about *E. quasihormaechei* ST873 as a high-risk clone for acquiring multidrug resistance, particularly carbapenemase resistance.

In summary, we demonstrated that the IncX3 *bla*_NDM-7_–carrying plasmids most probably derived of IncX3 *bla*_NDM-5_–carrying plasmids that were derived from IncX3 *bla*_NDM-1_–carrying plasmids and contain several additional features, including IS*5*, IS*Kox3*, and Tn*5403*. Despite the high transfer frequency (*14*) and low fitness cost (*15*) of IncX3 plasmids, their implication in the dissemination of *bla*_NDM_ remain unequal between Enterobacterales species. The strong association between IncX3 *bla*_NDM-7_–carrying plasmids and ECC underscores the need for enhanced surveillance to monitor spread of antimicrobial resistance.

Appendix 1 Additional information on emergence and polyclonal dissemination of *bla*_NDM-7_–carrying InX3 plasmid in *Enterobacter cloacae* complex, France, 2021–2023.

Appendix 2 Sequence data from a study of emergence and polyclonal dissemination of *bla*_NDM-7_–carrying InX3 plasmid in *Enterobacter cloacae* complex, France, 2021–2023.
