# Supplementary material for: Emergence and Polyclonal Dissemination of blaNDM-7–Carrying InX3 Plasmid in Enterobacter cloacae Complex, France, 2021–2023
Source: Emerg Infect Dis. 2025 Oct;31(10):1998–2002. doi: 10.3201/eid3110.250830 (PMC12483117; doi:10.3201/eid3110.250830)
Supplement: Appendix 1 — Additional information on emergence and polyclonal dissemination of blaNDM-7–carrying InX3 plasmid in Enterobacter cloacae complex, France, 2021–2023. [file 25-0830-Techapp-s1.pdf]

Article DOI: https://doi.org/10.3201/eid3110.250830

EID cannot ensure accessibility for supplementary materials supplied by authors. Readers who have difficulty accessing supplementary content should contact the authors for assistance.

Emergence and Polyclonal Dissemination of *bla*$_{NDM-7}$–Carrying InX3 Plasmid in *Enterobacter cloacae* Complex, France, 2021–2023

Appendix 1

Material and Methods

Data collection

The French National Reference Center (F-NRC) for Antimicrobial Resistance (Kremlin-Bicêtre, France) receives *Carbapenem-resistant Enterobacterales* (CRE) isolates voluntarily submitted by clinical microbiology laboratories from metropolitan France, overseas territories, and international regions for expert analysis of the carbapenem resistance mechanisms. These isolates originate from a variety of clinical samples, including urine, blood cultures, and rectal swabs.

Bacterial identification was performed using MALDI-TOF MS (Bruker Daltonics, Bremen, Germany). Carbapenemase production was assessed using the NG-Carba5 test (NG Biotech, Guipry, France) as recommended by the manufacturer instructions (*1*).

From January 1st, 2021 to December 31th, 2023, all NDM-producing isolates received at the FNRC for Antimicrobial Resistance were included in this study (n = 3365).

Whole Genome Sequencing (WGS) and genomic analysis

WGS was performed on all NDM-producing isolates included in this study using NextSeq500 sequencing technology (Illumina Inc., San Diego, CA, USA) with a 2x150-bp paired-end approach. Reads were assembled using Shovill v1.1.0 (https://bio.tools/shovill) and SPAdes v3.14.0. First, WGS data were used to confirm the bacterial species, using Centrifuge Taxonomic Classifer 1.0.3 (https://github.com/chienchi/kbase-centrifuge).Multilocus sequence typing (MLST) was performed using the MLST server v2.0

with automated analysis through a personal pipeline. Resistance genes and carbapenemase variants were identified using in-house bioinformatics pipelines, Resfinder 4.6.0 (http://genepi.food.dtu.dk/resfinder), CARD (https://card.mcmaster.ca), in conjunction with a comprehensive resistance database maintained by the F-NCR.

Long-Read Sequencing

To better understand the structures of *bla*$_{NDM-7}$–carrying plasmids and assess whether *bla*$_{NDM}$7-carrying plasmids shared similarities with those hosting other *bla*$_{NDM}$ variants, we selected 59 isolates belonging to the *Enterobacter cloacae* complex (ECC), including 30 NDM-7, 11 NDM-1 and 18 NDM-5 producers (Appendix 1 Figure 3). These 30 NDM-7–producing isolates were chosen to cover all replicases identified by short-read sequencing analysis (Appendix 2, https://wwwnc.cdc.gov/EID/article/31/10/25-0830-App2.xlsx). Long-read sequencing was performed using the Oxford Nanopore MinION platform (Oxford Nanopore Technologies, Oxford, UK), as previously described (*2*). MinION long-reads were assembled using Trycycler v0.5.5. and corrected with Illumina^{Ò} reads using Polypolish v0.6.0.

The sequencing data are available under GenBank accession numbers (Appendix 2).

Plasmids characterization

Plasmid content was assessed using the PlasmidFinder 2.1 database. Plasmid detection was performed on the entire collection of isolates using short-read sequencing data (Illumina). Additionally, for a subset of isolates, long-read sequencing (Nanopore) was used to enable precise plasmid characterization. Then, CLC Genomic Workbench v12,0 (QIAGEN, les Ulis, France), RAST v2.0 server (https://rast.nmpdr.org/) and ISFinder (https://isfinder.biotoul.fr) were used to annotate the genome and identify insertion sequences. The visualization of plasmids was achieved using Proksee software (https://proksee.ca).

Determination of the close genetic environments of blaNDM-7, blaNDM-5 and blaNDM-1 localized on IncX3 plasmids

To determine the genetic environments of the different *bla*$_{NDM}$ genes localized on IncX3 plasmids, we selected isolates 289A7 and 382A5 as representatives of *bla*$_{NDM-7}$, 386G2 and 370H10 as representatives of *bla*$_{NDM-5}$, and 383C1 as representative of *bla*$_{NDM-1}$ (Figure 3). By mapping the reads onto these representative strains, the same genetic environments were confirmed for the other isolates sequenced by long-read technology.

Evolution of NDM variants from 2021 to 2023

NDM-1 was the most prevalent variant. However, its proportion declined from 56.4% in 2021 to 43.7% in 2023. In contrast, the ratio of NDM-5 increased from 31.0% in 2021 to 42.1% in 2023. Regarding the two others prevalent NDM variants, (i) NDM-7 remained stable over time representing 8% (6.9% to 8.7%) of NDM producers, and (ii) NDM-14, which emerged in 2022 rose from 1.0% of the NDM-producers in 2021 to 4.6% in 2022 to stabilized at 4.1% in 2023 (Figure 1, panel A).

Distribution of species among NDM-producing Enterobacterales (2021–2023)

NDM-type carbapenemases have been identified in 10 different Enterobacterales genus including 368 *Citrobacter* spp. (342 *C. freundii*, 4 *C. koseri*, 5 *C. farmeri*, 6 *C. amalonaticus*, 7 *C. braakii* and 4 other *Citrobacte*r spp.), 538 *Enterobacter cloacae* complex, 1093 *Escherichia coli*, 1308 *Klebsiella spp.* (1,226 *K. pneumoniae*, 67 *K. oxytoca*, 15 *K. aerogenes*), 17 *Morganella morganii*, 14 *Proteus spp.* (12 *P. mirabilis*, 1 *P. penneri* and 1 *P. vulgaris*), 9 *Providencia* spp. (3 *P. stuartii* and 6 *P. rettgeri*), 12 *Serratia marcescens*, and 3 others Enterobacterales species (1 *Hafnia alvei*, 1 *Kluyvera cryocrescens* and 1 *Leclercia adecarboxylata*).

Phylogenetic relatedness, isolation date, isolates' origin and single nucleotide matrix of the most prevalent clones NDM-7–producing Enterobacter cloacae complex (ST873, ST135, ST145 and ST683)

Phylogenetic analysis with single nucleotide polymorphisms determination were performed on the four major STs displaying several isolates to identify any clonal dissemination of single strain. For ST873 *E. quasihormaechei*, four distinct clones were identified among the 23 isolates. In the case of ST145 *E. hoffmannii*, four distinct clones were also observed among the 11 isolates. The 11 isolates of ST135 *E. hoffmannii* corresponded to the dissemination of two different clones, and the 10 isolates of ST683 *E. hoffmannii* were more diverse since the correspond to five unrelated clones.

References

1. Bernabeu S, Bonnin RA, Dortet L. Comment on: Comparison of three lateral flow immunochromatographic assays for the rapid detection of KPC, NDM, IMP, VIM and OXA-48 carbapenemases in Enterobacterales. J Antimicrob Chemother. 2023;78:314–7. PubMed https://doi.org/10.1093/jac/dkac381

2. Girlich D, Bonnin RA, Proust A, Naas T, Dortet L. Undetectable production of the VIM-1 carbapenemase in an *Atlantibacter hermannii* clinical isolate. Front Microbiol. 2021;12:741972. PubMed https://doi.org/10.3389/fmicb.2021.741972

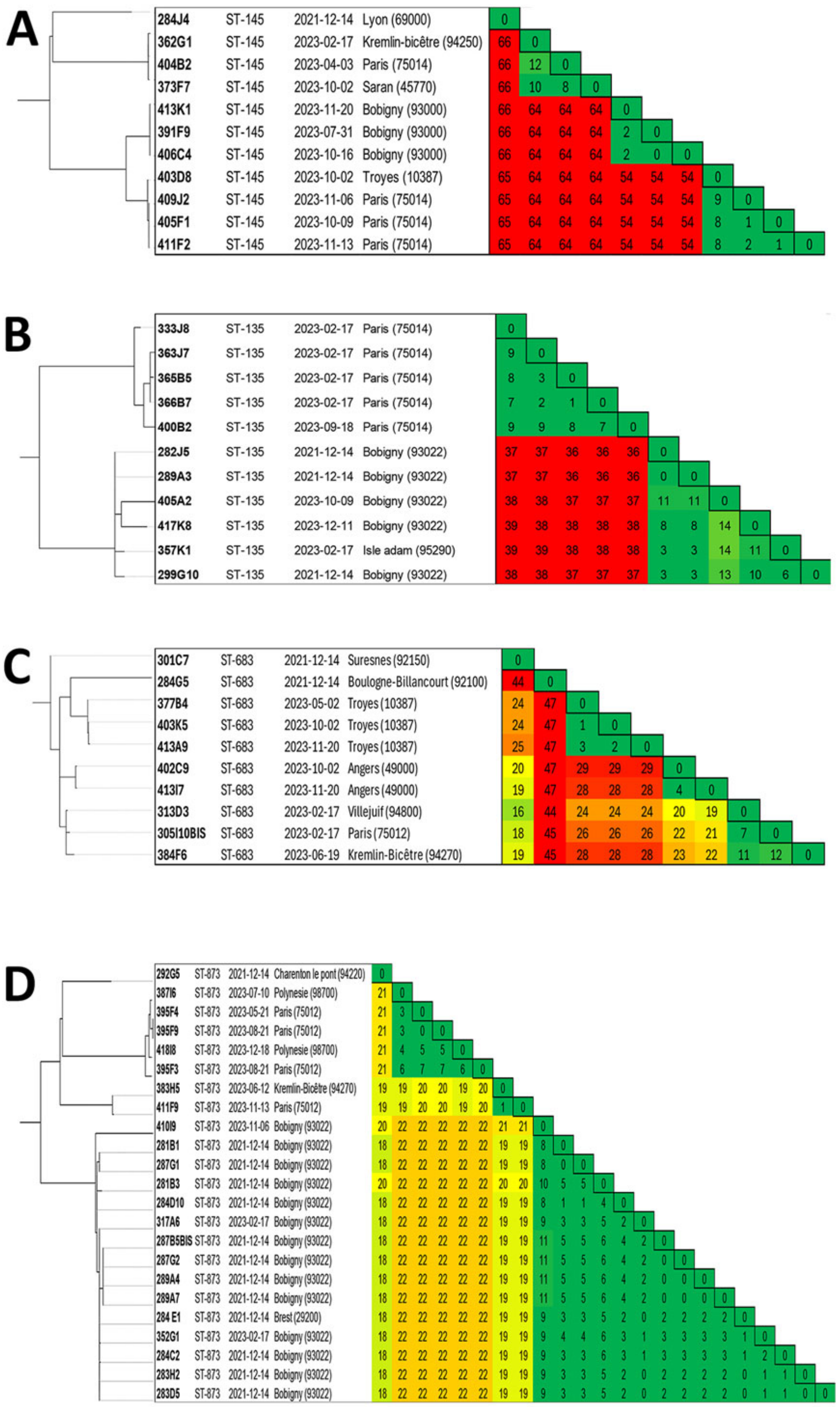

Appendix 1 Figure 1. Phylogenetic relatedness, isolation date, isolates' origin and single nucleotide matrix of the most prevalent clones NDM-7-producing Enterobacter cloacae complex (ST-873, ST-135, ST-145 and ST-683).

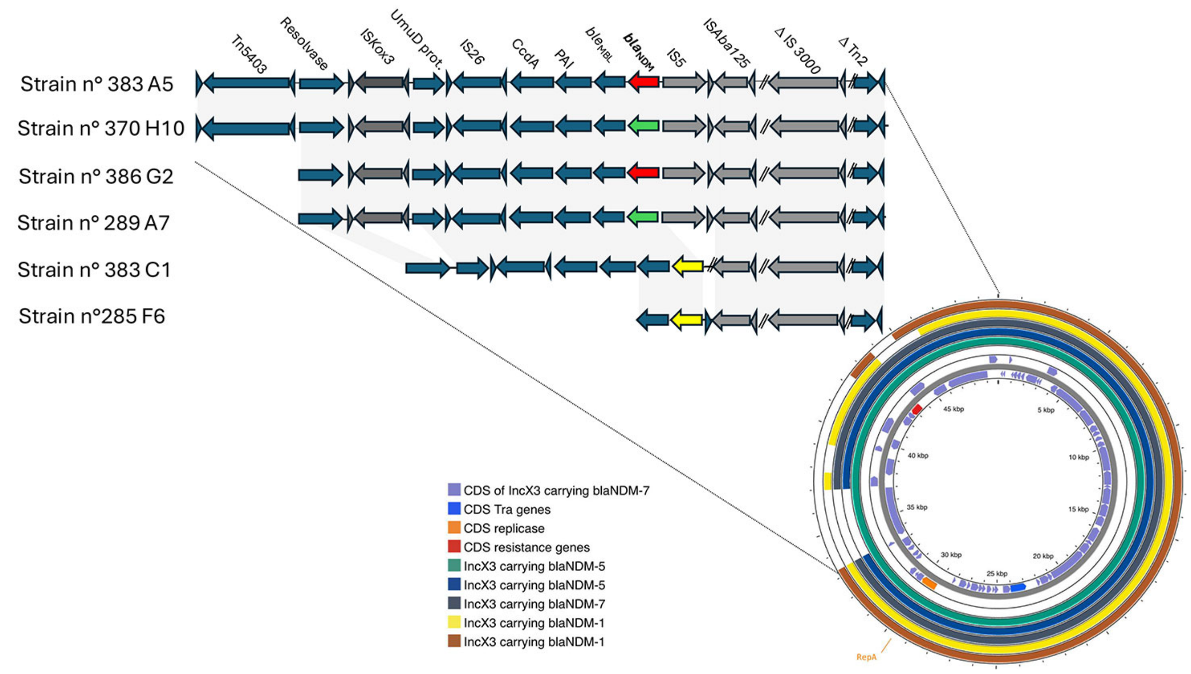

Appendix 1 Figure 2. Close genetic environment of *bla*$_{NDM-1}$, *bla*$_{NDM-5}$ and *bla*$_{NDM-7}$ and circular representation of IncX3 *bla*$_{NDM-1}$-, *bla*$_{NDM-5}$– and *bla*$_{NDM-7}$–carrying plasmids. CDS: coding sequence. The representation was created using Proksee software. On the plasmid representation resistance genes are represented by red arrows, the *tra* genes implicated in plasmid self-conjugation are colored in blue, and replicase in orange, the other CDS are represented by purple arrows. In close genetic environment, *bla*$_{NDM-1}$, *bla*$_{NDM-5}$, and *bla*$_{NDM-7}$ are represented by yellow, green and red arrows, respectively. Nucleotide sequences with >99% identity are highlighted in gray.

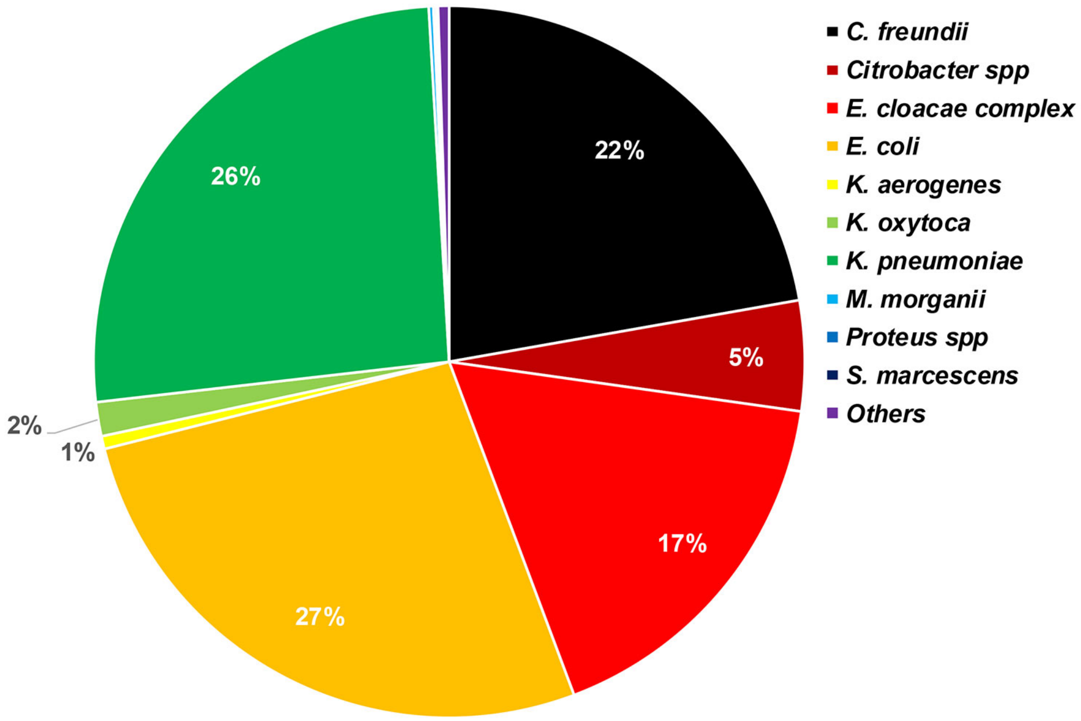

Appendix 1 Figure 3. Species distribution among the 2,393 Enterobacterales carrying an IncX3 encoding replicase gene, 11.9% of the 20,028 multidrug-resistant Enterobacterales genomes (since 2022) of the F-NRC database.
